# Design and Hardware Implementation of a New Chaotic Secure Communication Technique

**DOI:** 10.1371/journal.pone.0158348

**Published:** 2016-08-22

**Authors:** Li Xiong, Yan-Jun Lu, Yong-Fang Zhang, Xin-Guo Zhang, Parag Gupta

**Affiliations:** 1 School of Mechanical and Precision Instrument Engineering, Xi’an University of Technology, Xi’an, 710048, China; 2 School of Physics and Electromechanical Engineering, He’xi University, Zhang’ye, 734000, China; 3 School of Printing, Packaging Engineering and Digital Media, Xi’an University of Technology, Xi’an, 710048, China; 4 School of Information Science and Engineering, Lan’zhou University, Lan’zhou, 730000, China; 5 Robert R. McCormick School of Engineering and Applied Science, Northwestern University, Evanston, IL, 60208, United States of America; Lanzhou University of Technology, CHINA

## Abstract

In this paper, a scheme for chaotic modulation secure communication is proposed based on chaotic synchronization of an improved Lorenz system. For the first time, the intensity limit and stability of the transmitted signal, the characteristics of broadband and the requirements for accuracy of electronic components are presented by Multisim simulation. In addition, some improvements are made on the measurement method and the proposed experimental circuit in order to facilitate the experiments of chaotic synchronization, chaotic non-synchronization, experiment without signal and experiment with signal. To illustrate the effectiveness of the proposed scheme, some numerical simulations are presented. Then, the proposed chaotic secure communication circuit is implemented through analog electronic circuit, which is characterized by its high accuracy and good robustness.

## 1. Introduction

Since the American scholars Pecora and Carroll proposed a type of drive-response synchronization scheme [1], researchers on chaos have been greatly inspired. As are well known, the chaotic signals have the following characteristics: sensitive dependence on the initial conditions, unpredictability, similarity to the noise, and difficulty to be deciphered. Therefore, it is especially suitable to be applied to the secure communication field. Chaotic secure communication is one of the most remarkable phenomena in the present physical field, and it is one of the most promising applications in chaotic circuits. What presented is very different from the previous findings. Chaotic secure communication can be shown to correspond to the phenomenon of resonance and mode locking in nature, and it also utilizes the ergodic property that is an important feature of chaos. Thus it has caused huge attention from various industries.

Several works can be found in literature about chaotic secure communications. As early as 1993, Cuomo and Oppenheim implemented the masking secure communication scheme of Lorenz system [2, 3]. In 1996, Milanović and Zaghloul put forward an improved scheme of chaotic masking secure communication [4]. Later the chaotic secure communication circuit was implemented by electronic components [5] based on Lorenz system according to work [4]. With the fast development of chaotic secure communication, work [6] approached the problem of synchronization of chaotic systems from the perspective of Generalized Hamiltonian systems. Then, that approach was applied to the chaotic secure communication system based on two Chua’s oscillators and an experimental implementation was presented by using CCII plus s [7]. In 2009, Arman et al. proposed a fractional chaotic communication method using an extended fractional Kalman [8]. The proposed stabilization conditions were used in work [9] to design a linear-state-observer for the secure communization of a wide class of discrete-time hyperchaotic system via a scalar transmitted signal. In work [10] a new chaotic secure communication scheme was proposed based on chaotic Duffing oscillators and frequency estimation for the transmission of binary-coded messages. In work [11] a chaotic modulation secure communication scheme was proposed based on improved Chua's circuit. In work [12], the design and implementation of adaptive Generalized Projective Synchronization (GPS) are studied between two chaotic circuits (master and slave) via a scalar transmitted signal and by a new method not requiring the same structure of master and slave circuits. In addition, it is well known that chaotic attractor can be properly used in secure communication system. Particularly, the chaotic systems composed of multi-scroll attractors are much preferred to the double-scroll attractors because they offer more dynamical complexity [13–19]. In order to transmit high-speed data, the chaotic attractors should operate at high frequency. However, it is difficult to enhance the frequency response of analog realizations of chaotic oscillator when it is designed with integrated circuit technology. Besides, FPGA based realization emerged as a solution to observe attractors at high frequency. In the paper [20], it has been shown that by using FPGAs one can realize multi-scroll chaotic oscillators that have better behavior than by using active devices like operational amplifiers. In work [21] a FPGA realization of a chaotic communication system was proposed to be applied to image processing. Synchronization can also be extended to complex topologies with multi-scroll attractors [22, 23].

Despite the fact that chaotic secure communication has advantages of strong real-time performance and high security performance, the study of chaotic secure communication is still in the phase of laboratory research [24–27]. Many problems still need to be solved in the study of chaotic secure communication. On one hand, the contradiction between the confidential party and the broken party often leads to a complex circuit implementation [28–37]. Therefore, because most researchers still focus on the study of chaos theory in numerical simulation, there is a certain deviation of the physical circuit system. On the other hand, some shortcomings in terms of fidelity and safety of most chaotic secure communication schemes could not guarantee a system retained synchronization in theory under large signal conditions. In addition, being lack of optimization and improvement on the experimental circuit and measurement method, the comprehensive statements are precluded from being drawn from experimental results. In general, the three main types of chaotic secure communication are chaotic masking [4], chaotic modulation [25] and chaotic shift keying [38]. The chaotic modulation method has the following advantages compared with the other two types: first, it is used to hide the whole range of chaotic signal spectrum information allowing for a wide spectrum of feature; second, it is more sensitive to parameter variation, thereby enhancing the confidentiality. In this paper, a chaotic modulation method is proposed to establish secure communication based on an improved Lorenz chaotic optimization circuit.

The main contributions of this paper include: In Section 2, the active control method is adopted to control the improved Lorenz chaotic system. With active control method, the synchronization error system can be asymptotically stabilized at the origin. In Section 3, the chaotic modulation secure communication scheme is proposed based on synchronization of chaos between a transmitter and a receiver linked by a transmission channel. In the proposed scheme, with an improved Lorenz system as chaos generator, the chaotic modulation secure communication is implemented by using some electronic components containing analog multipliers, operational amplifiers, resistors, and capacitors. In Section 4, because the implementation of the Lorenz circuit needs analog multipliers, higher accuracy requirements on the parameters of the electronic components is necessary. Therefore, the intensity limit and stability of the transmitted signal, the broadband characteristic, and the accuracy requirements of electronic components are presented for the first time by Multisim simulation. In Section 5, some improvements on the experimental circuit and measurement method of the proposed secure communication circuit are introduced. Experiments of chaotic synchronization, experiments of chaotic non-synchronization, experiments without signal, and experiments with signal are presented to verify the comprehensive performance of the proposed scheme. Some numerical simulations are presented to verify the feasibility and effectiveness of the scheme.

Moreover, the study on chaotic circuit is the premise and foundation of the physical circuit verification. Also, it can deepen the understanding of chaos and expand its application scope. In Section 6, the proposed secure communication circuit is implemented in an analog electronic circuit. The analog circuit implementation results match the Multisim and Matlab simulation results. Such measurement method and experimental results have not been reported previously. Thus, the results of this work are quite valuable in practical application. Finally, conclusions and discussions are presented in Section 7. The proposed scheme is not restricted to the Lorenz system and, in fact, can also be used in other chaotic systems.

## 2. Synchronization of Improved Lorenz Chaotic System

The basic Lorenz equation is described as follows:

$$\left\{ \begin{array}{l} \dot{x}=\sigma (y-x)\\ \dot{y}=\rho x-y-xz\\ \dot{z}=xy-\beta z \end{array}\right.$$ (1)

When choosing *σ* = 10, *ρ* = 28, and *β* = 8/3, system (1) is chaotic. However, the numerical solutions of the basic Lorenz equation are not able to be implemented by using general circuit components. Thus, in practice, these variables often needs to be adjusted properly. The introduction of new variables is described as follows:

$$\begin{array}{ccc} u=\frac{x}{10}, & v=\frac{y}{10}, & w=\frac{z}{30} \end{array}$$ (2)

Substituting the specific parameter values, eq (1) becomes

$$\left\{ \begin{array}{l} \dot{x}=-10x+10y\\ \dot{y}=28x-y-30xz\\ \dot{z}=3.3xy-(8/3)z \end{array}\right.$$ (3)

This is the improved Lorenz chaotic system. It fully conforms to the requirements of the circuit design in practical applications.

The drive system is provided for

$$\left\{ \begin{array}{l} {\dot{x}}_{1}=a(x_{\text{2}}-x_{1}),\\ {\dot{x}}_{2}=bx_{1}-x_{2}-30x_{1}x_{3},\\ {\dot{x}}_{3}=3.3x_{1}x_{2}-cx_{3}, \end{array}\right.$$ (4)

When choosing *a* = 10, *b* = 28, and *c* = 8/3, system (4) is chaotic, and the response system is described as follows:

$$\left\{ \begin{array}{l} {\dot{y}}_{1}=a(y_{2}-y_{1})+u_{1},\\ {\dot{y}}_{2}=by_{1}-y_{2}-30y_{1}y_{3}+u_{2},\\ {\dot{y}}_{3}=3.3y_{1}y_{2}-cy_{3}+u_{3}, \end{array}\right.$$ (5)

where *u*_1_, *u*_2_, and *u*_3_ are the controllers [39]. When the synchronization error is defined as $\dot{e}=\dot{y}-\dot{x}$, then the synchronization error of systems (4) and (5) can be described as follows:

$$\left\{ \begin{array}{l} {\dot{e}}_{1}=a(e_{2}-e_{1})+u_{1},\\ {\dot{e}}_{2}=be_{1}-e_{2}-30y_{1}y_{3}+30x_{1}x_{3}+u_{2},\\ {\dot{e}}_{3}=3.3y_{1}y_{2}-3.3x_{1}x_{2}-ce_{3}+u_{3}, \end{array}\right.$$ (6)

The controller is constructed as follows:

$$\left\{ \begin{array}{l} u_{1}=-a(e_{2}-e_{1})-k_{1}e_{1}\\ u_{2}=-30x_{1}x_{3}+30y_{1}y_{3}+e_{2}-be_{1}-k_{2}e_{2}\\ u_{3}=ce_{3}+3.3x_{1}x_{2}-3.3y_{1}y_{2}-k_{3}e_{3} \end{array}\right.$$ (7)

where *k*_*i*_ > 0(*i* = 1,2,3), for controlling the speed of synchronization.

Substituting Eqs (7) into (6), the following is obtained:

$$\left\{ \begin{array}{l} {\dot{e}}_{1}=-k_{1}e_{1},\\ {\dot{e}}_{2}=-k_{2}e_{2},\\ {\dot{e}}_{3}=-k_{3}e_{3}, \end{array}\right.$$ (8)

To facilitate the control design, the Lyapunov function *V* [40, 41] is defined as follows:

$$V=(e_{1}^{2}+e_{2}^{2}+e_{3}^{2})/2,$$ (9)

Obviously, *V* is positively definite. It follows from eq (9) that,

$$\begin{array}{ll} \dot{V} & =e_{1}{\dot{e}}_{1}+e_{2}{\dot{e}}_{2}+e_{3}{\dot{e}}_{3}\\ & =e_{1}(-k_{1}e_{1})+e_{2}(-k_{2}e_{2})+e_{3}(-k_{3}e_{3})\\ & =-k_{1}e_{1}^{2}-k_{2}e_{2}^{2}-k_{3}e_{3}^{2} \end{array}$$ (10)

Then, $\dot{V}=-k_{1}e_{1}^{2}-k_{2}e_{2}^{2}-k_{3}e_{3}^{2}\leq 0$ is obtained. Thus, $\dot{V}$ is negatively semidefinite.

According to Lyapunov stability theory, if *V* is positively definite and $\dot{V}$ is negatively semidefinite, then the system is consistent and stable at the origin of the equilibrium state. Therefore, the synchronization error system (6) is asymptotically stable at the origin. That is, $\underset{t\to \infty }{\text{lim}}|e(t)|\to 0$. This proves that the active synchronization between the drive system and the response system is achieved.

**Fig 1:**
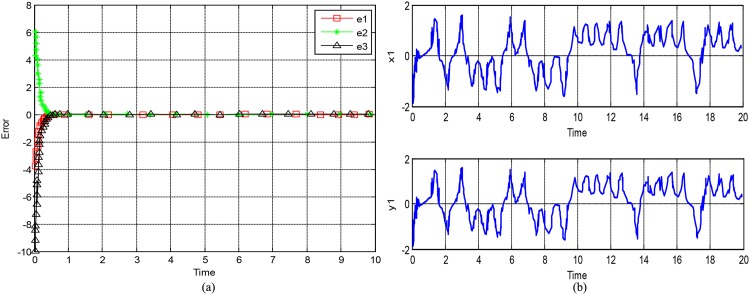
Synchronization error curve and waveform. For **(a)** synchronization error, for **(b)** synchronized waveform of *x*_1_ − *y*_1_.

In the following simulation, the initial values of the drive system are chosen as *x*_1_(0) = 2, *x*_2_(0) = −3, and *x*_3_(0) = 6. The initial values of the response system are chosen as *y*_1_(0) = −3, *y*_2_(0) = 5, and *y*_3_(0) = −4. The control gains are chosen as *k*_1_ = *k*_2_ = *k*_3_ = 10. The synchronization error curves are shown in Fig 1(a). As can be seen from the figures, for less than 1 second, the synchronization errors *e*_1_, *e*_2_, and *e*_3_ can be asymptotically stabilized at the origin. Fig 1(b) shows the timing diagram of *x*_1_ − *y*_1_. The waveforms of the two systems are shown to be the same, and the active control synchronization is implemented. The method is simple and practical, and the synchronization time is very short. As is well known in practical application, the smaller the control signal is, the more easily the hardware circuit of the control process is implemented. Therefore, the proposed scheme is easier to be implemented in the hardware circuit because of its low control signal and low cost.

## 3. Proposed Chaotic Secure Communication Circuit Scheme

The implementation of chaotic synchronization [42] solves a large and difficult problem of chaotic secure communication technology [43,44]. Although the chaotic system is especially suitable for secure communication, the synchronization between a transmitting system and a receiving system should be completed in order to achieve Lorenz chaotic secure communication. Through analysis and optimization of the improved Lorenz circuit, inverter and adder were added, thus realizing the evolution from freedom chaos after chaotic synchronization to secure communication. Here, the chaotic secure communication is implemented by using chaotic modulation.

**Fig 2:**
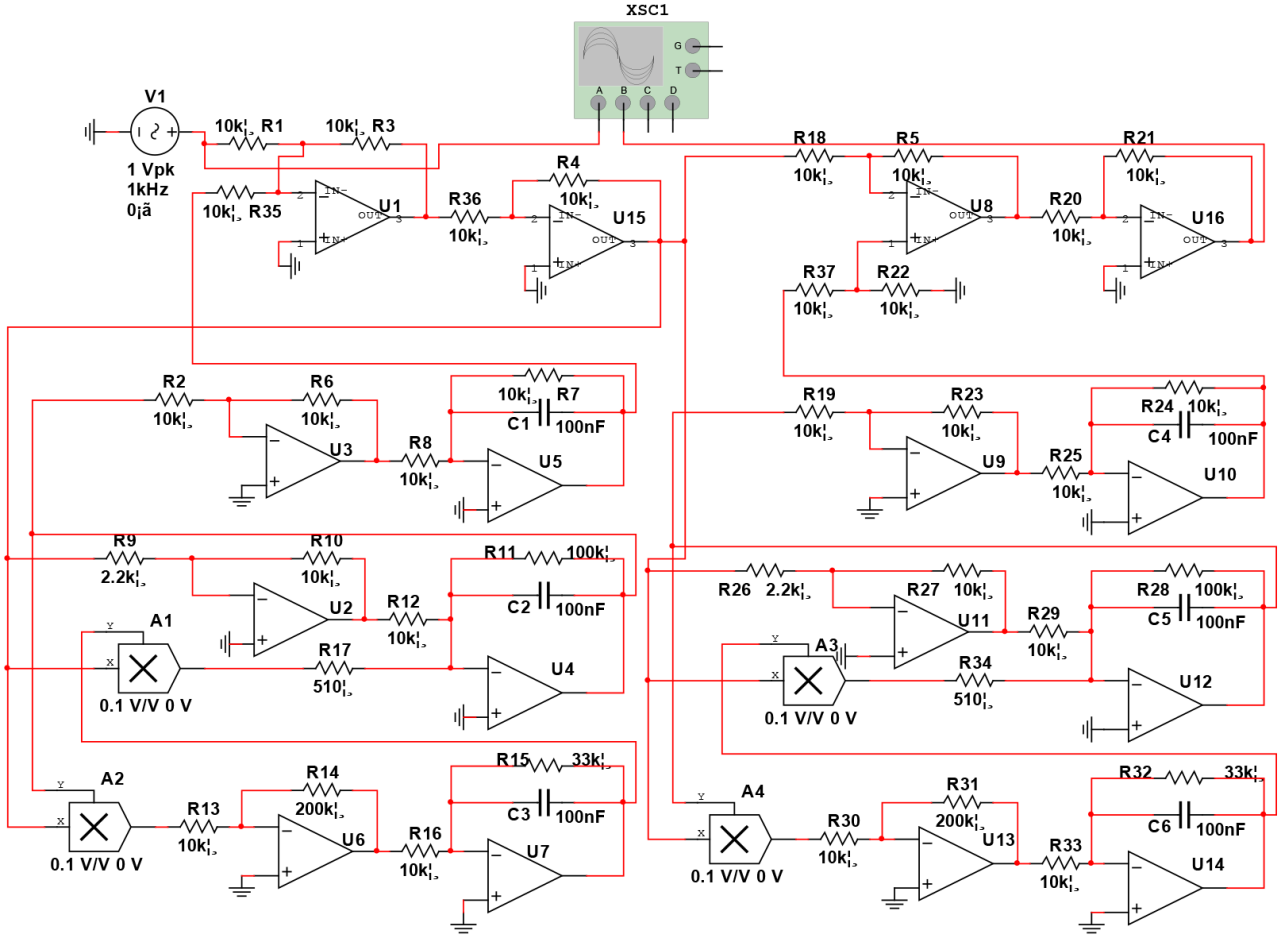
Chaotic secure communication optimization circuit schematic.

The preceding active control scheme of synchronization is applied to establish a chaotic secure communication circuit. The chaotic modulation secure communication circuit schematic based on improved Lorenz system is shown in Fig 2 by using Multisim software. The left side of the circuit is the transmitting system. The right side of the circuit is the receiving system. The uppermost operational amplifiers of the transmitting system are designed as a modulator. The modulation circuit consisting of an inverting adder and an inverter plays a signal superposition function. Its output signal is transmitted to the receiving system through a communication channel (wired or wireless). The chaotic circuit of the receiving system is the same as the transmitting system. The uppermost operational amplifier of the receiving system are designed as a demodulator, which is composed of a same-phase adder. Its input is chaotic signal, and its output is the error signal of two chaotic signals, that just happens to be the transmitted signal of the transmitter, thus completing the chaotic secure communication.

The whole process of the proposed chaotic secure communication can be expressed as follows: suppose *x*(*t*) is a transmitted signal, *s*(*t*) is a chaotic signal, and *y*_1_(*t*) is superimposed signal. Then the superimposed signal is described as:

$$y_{1}(t)=x(t)+s(t)$$ (11)

−*y*_1_(*t*) is the output from the inverting adder of modulation circuit, given by

$$-y_{1}(t)=x(t)+s(t)$$ (12)

The output after the inverter becomes

$$y_{2}(t)=-y_{1}(t)$$ (13)

The superimposed signal is described as

$$y_{1}(t)=-x(t)-s(t)$$ (14)

*y*_2_(*t*) is the output signal from the modulation circuit, given that

$$y_{2}(t)=x(t)+s(t)$$ (15)

*m*(*t*) is a received signal, given that

$$m(t)=-[s(t)-y_{2}(t)]=x(t)$$ (16)

In this way, the receiving system maintains synchronized with the transmitting system more easily, and the robustness of the circuit is maintained. Such an approach can prevent the effective information from being intercepted in the process of communication.

## 4. Multisim Simulation Experiments

**Fig 3:**
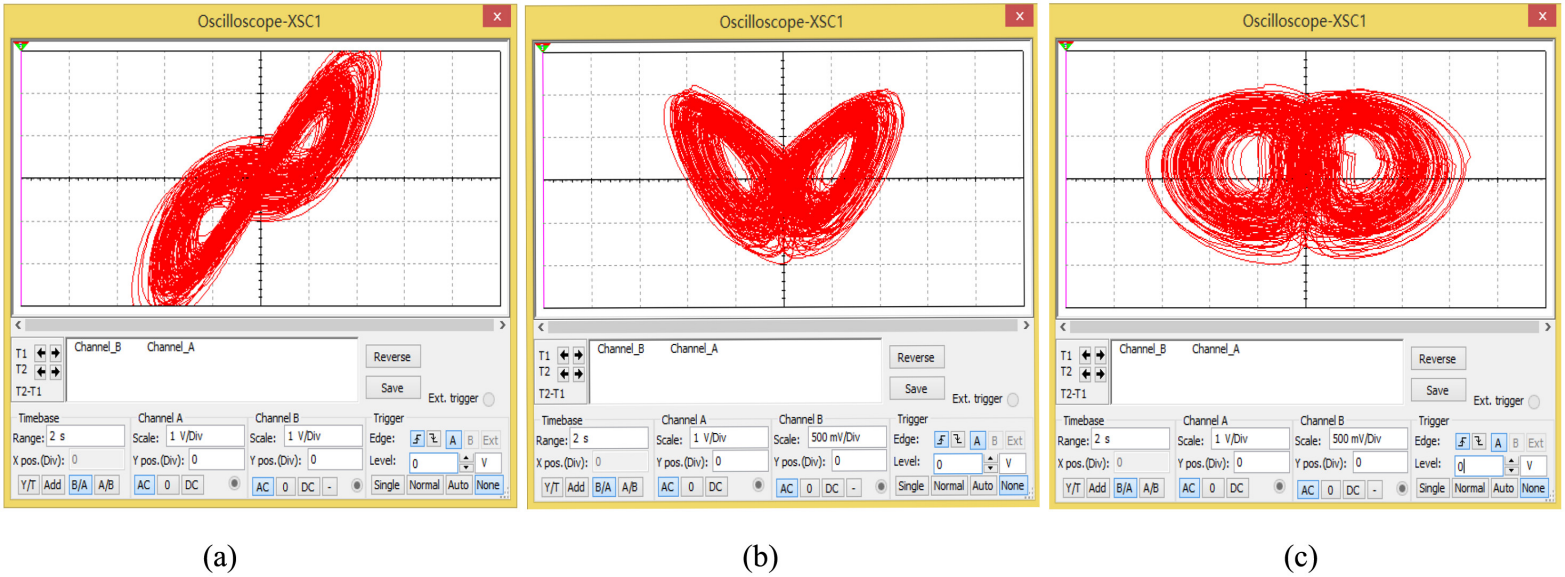
Chaotic phase diagram with Multisim. **(a)** *xy* phase diagram, **(b)** *xz* phase diagram, **(c)** *zy* phase diagram.

Before making the actual circuit, some simulations for the proposed chaotic secure communication optimization circuit by using Multisim software were conducted. The chaotic simulation phase diagrams of *xy*, *xz*, and *zy* are shown in Fig 3 by using Multisim software.

### 4.1. Synchronous Experiments

Here, the verification of whether two identical parameters of Lorenz optimization circuit can achieve the signal transmission and reception without distortion through synchronous experiments was conducted. The experimental circuit is shown in Fig 2, and the component parameters of the transmitting circuit are completely consistent with the receiving circuit.

**Fig 4:**
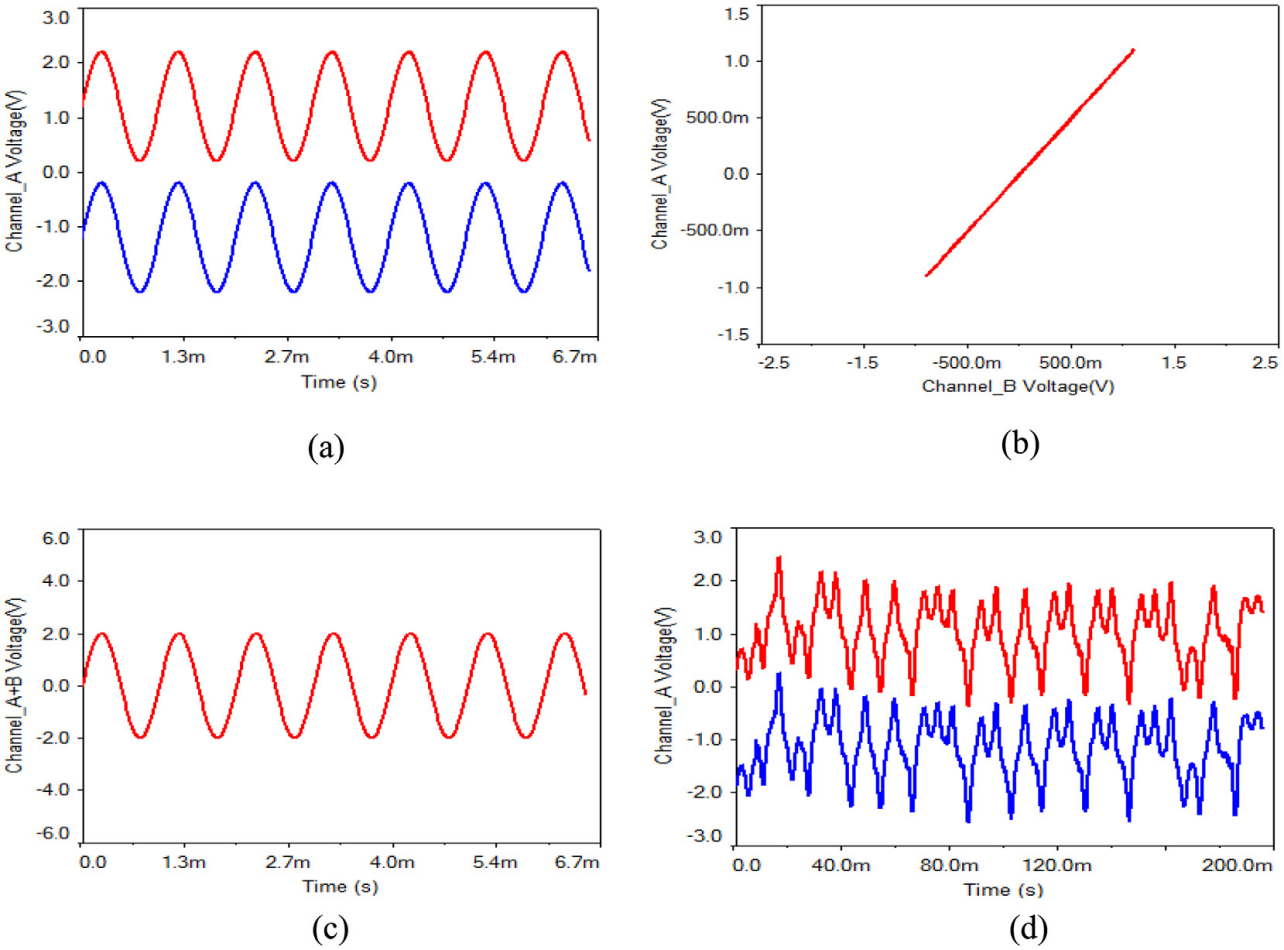
Waveforms of input sine wave with amplitude of 1 V and frequency of 1 kHz. **(a)** transmitting and receiving waveform, **(b)** synchronous phase diagram, **(c)** superimposed signal waveform, **(d)** carrier signal waveform.

**Fig 5:**
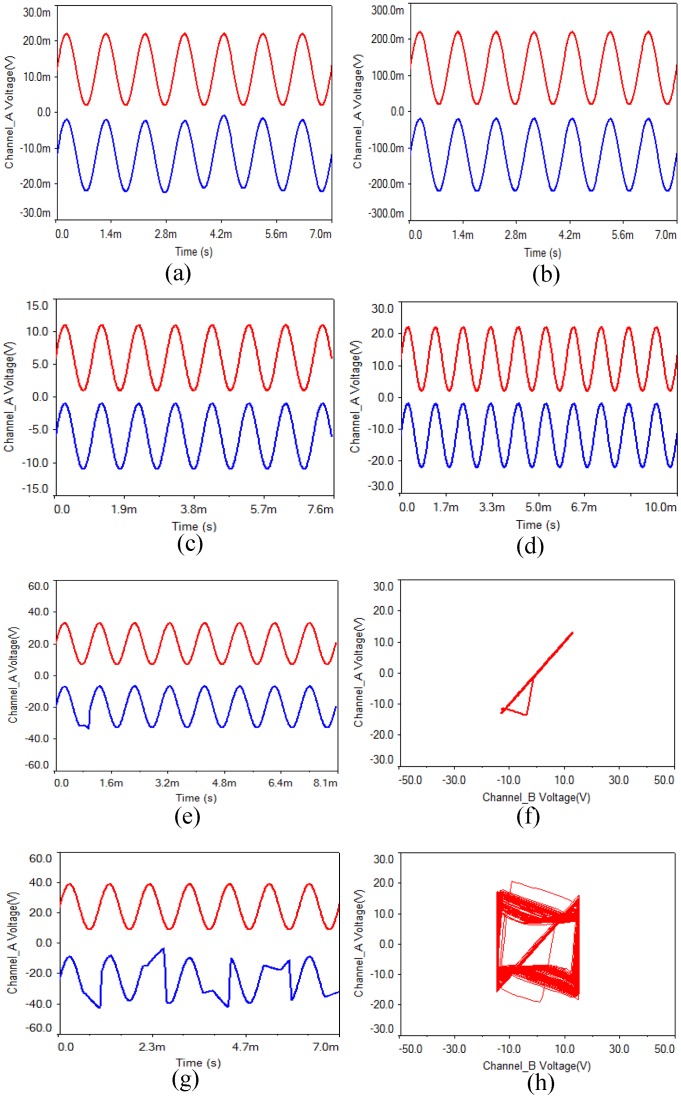
Waveforms of input sine wave with different amplitude. **(a)** 10 mV, **(b)** 100 mV, **(c)** 5 V, **(d)** 10 V, **(e)** 13 V, **(f)** synchronous phase diagram of 13 V, **(g)** 15 V, **(h)** phase diagram of 15 V.

**Fig 6:**
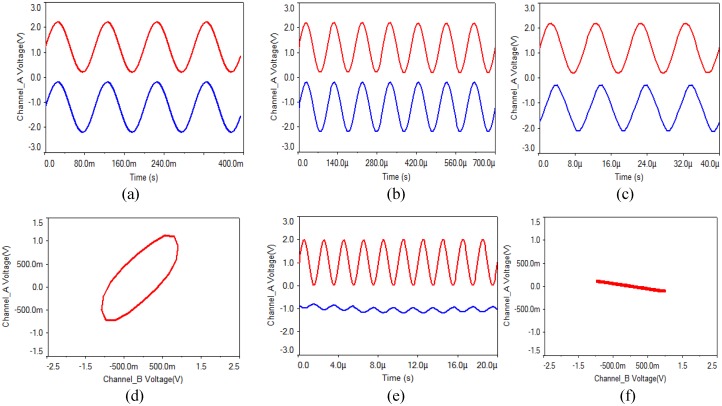
Waveforms of input sine wave with different frequency. **(a)** 10 Hz, **(b)** 10 kHz, **(c)** 100 kHz, **(d)** phase diagram of 100 kHz, **(e)** 500 kHz, **(f)** phase diagram of 500 kHz.

(1) Given an input sine wave with amplitude of 1 V and frequency of 1 kHz, the simulation results are shown as Fig 4. In order to verify that the proposed circuit can transmit various signals without distortion, square wave and triangular wave with amplitude of 1 V and frequency of 1 kHz were input. Simulation results show that, no matter what kind of signal is input, the two chaotic circuits can be completely synchronized if the component parameters of the transmitting circuit are entirely same as the receiving circuit. The modulation-demodulation signal waveform and the synchronous phase diagram of the receiver and the transmitter are shown in Fig 4(a), 4(b) and 4(c). Negligible distortion can be observed. The carrier signal waveform of the receiver and the transmitter is shown in Fig 4(d), and it is chaotic.

(2) In order to verify whether the proposed circuit has a choice for the intensity of various input signals, sine wave with frequency of 1 kHz and amplitude of 10 mV, 100 mV, 1 V, 5 V, 7 V, 10 V, 13 V, 15 V were input, and some simulation results are shown in Fig 5. From these waveforms, it is concluded that signal transmission distortion will appear when the signal amplitude reaches 13 V, as shown in Fig 5(e) and 5(f). When the signal amplitude reaches 15 V, the signal distortion is very obvious, as shown in Fig 5(g) and 5(h).

(3) In order to verify whether the proposed circuit has a choice for the input signal frequency, sine wave with amplitude of 1 V and frequency of 10 Hz, 100 Hz, 1 kHz, 10 kHz, 100 kHz, 500 kHz were input, and some simulation results are shown in Fig 6. From these waveforms, it is concluded that the proposed circuit can transmit the signal from 1 Hz to 50 kHz without distortion. When the signal frequency reaches 100 kHz, the signal distortion is very obvious, as shown in Fig 6(c) and 6(d). Therefore, it can be seen that the circuit is broadband.

### 4.2. Error Experiments

If an error exists in the parameter of a certain circuit component in the proposed chaotic secure communication circuit, whether or not the circuit can also keep synchronized is a question worthy to consider. In the experiments, we choose analog multiplier, capacitor, and operational amplifier to carry out the error analysis for the proposed chaotic secure communication circuit.

**Fig 7:**
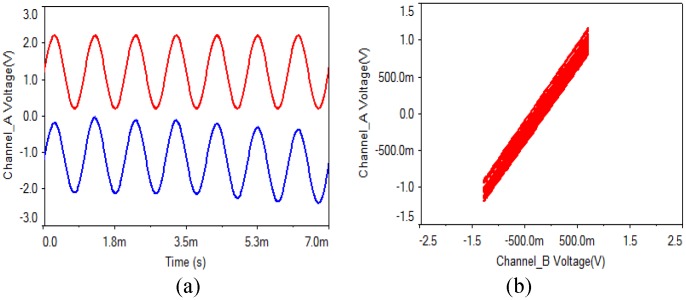
Analog multiplier of receiving circuit with 0.1% error. **(a)** transmitting and receiving waveform, **(b)** phase diagram.

(1) Analog multiplier: As is shown in Fig 2, the output gains of four analog multipliers are 0.1 V/V. In the experiments, when a multiplier parameter of the receiving circuit is chosen as 0.11 V/V, the waveform and the phase diagram are shown in Fig 7(a) and 7(b). What can be seen is that the synchronization can be implemented when a multiplier parameter of the receiving circuit has a small error of 0.1%, but there is a faint noise.

**Fig 8:**
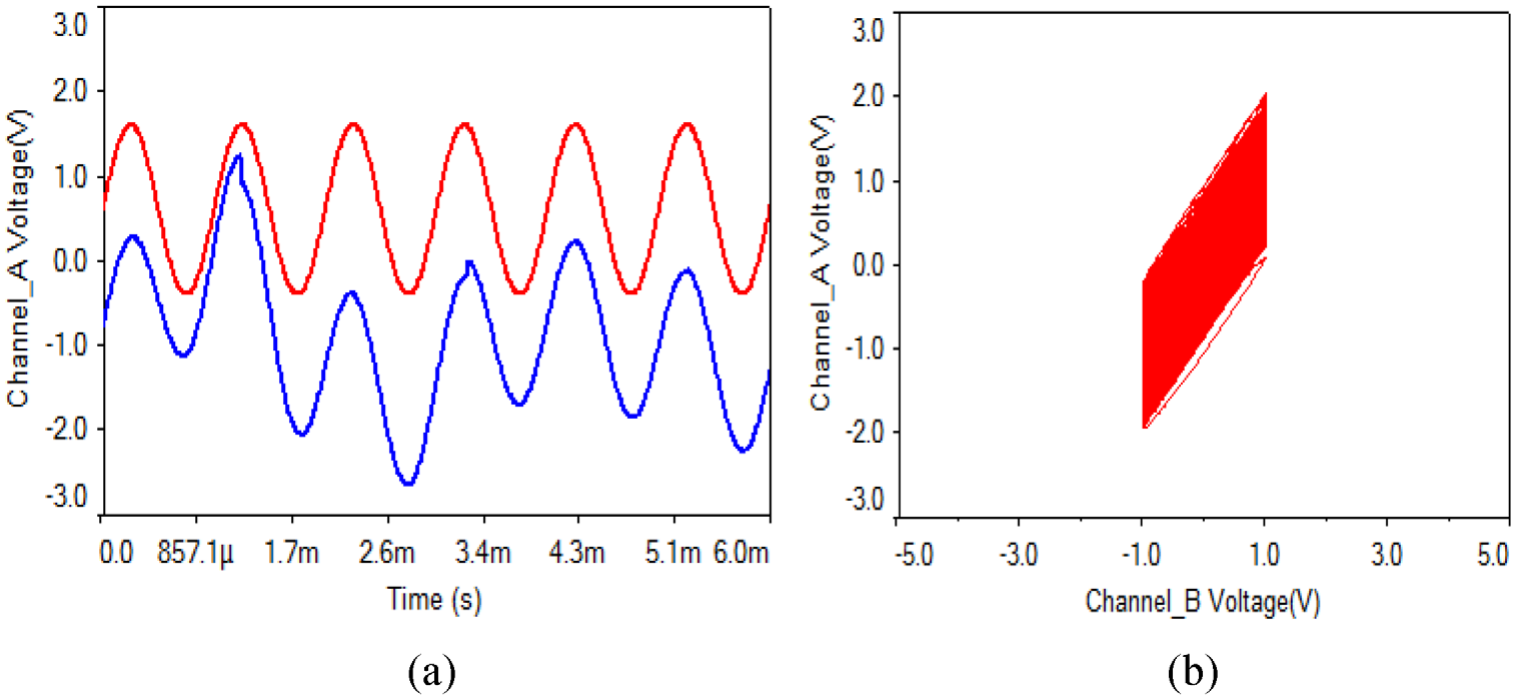
Analog multiplier of receiving circuit with 1% error. **(a)** transmitting and receiving waveform, **(b)** phase diagram.

Then, when a multiplier parameter of the receiving circuit is chosen as 0.2 V/V, the waveform and the phase diagram are shown in Fig 8(a) and 8(b). From the experimental results, what can be seen is that the synchronization can be still realized when an analog multiplier parameter of the receiving circuit has an error of 1%, but the noise is obvious.

**Fig 9:**
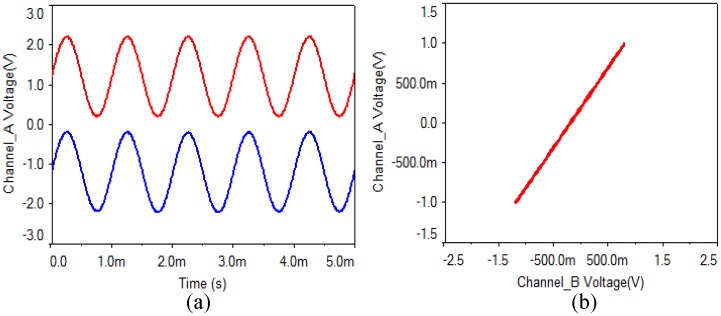
Capacitor of receiving circuit with 1% error. **(a)** transmitting and receiving waveform, **(b)** phase diagram.

**Fig 10:**
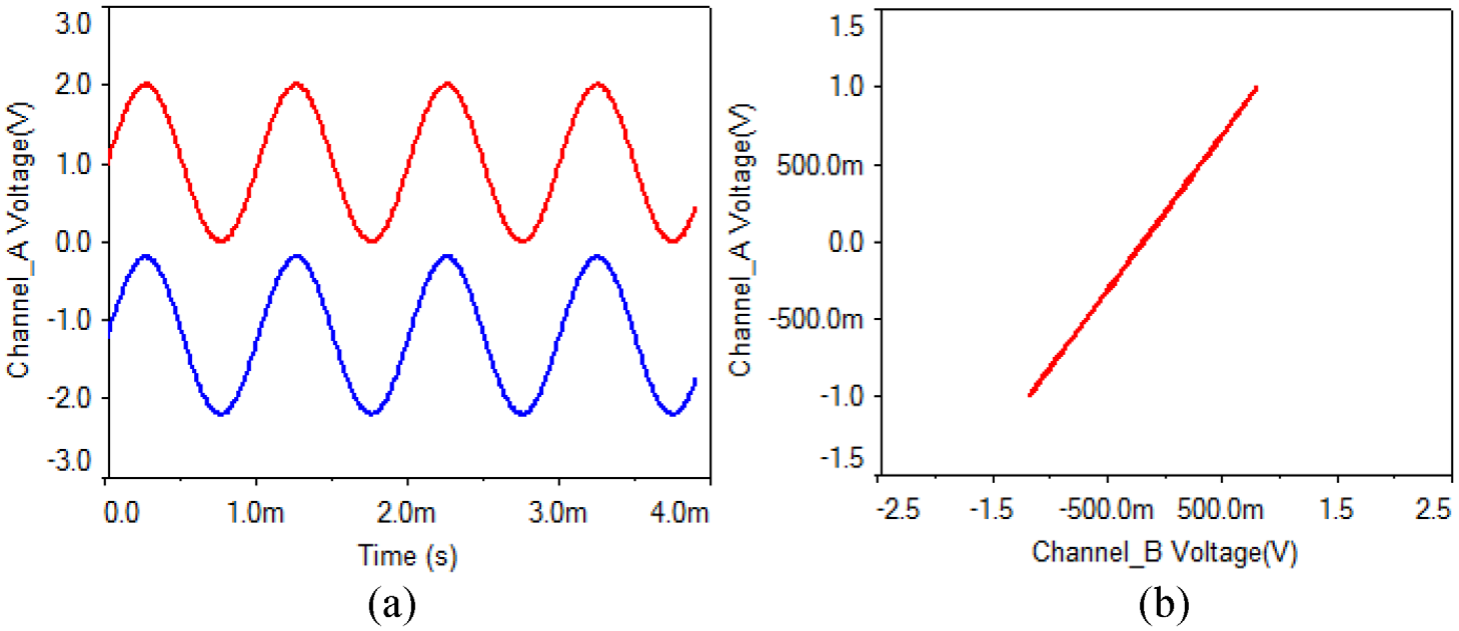
Operational amplifier of receiving circuit with 1% error. **(a)** transmitting and receiving waveform, **(b)** phase diagram.

(2) Capacitor: As is shown in Fig 2, the capacitor values of the transmitting circuit and the receiving circuit are 100 nF. In the experiments, when the value of C4 is chosen as 101 nF, the waveform and the phase diagram are shown in Fig 9(a) and 9(b). What can be seen is that the synchronization can be implemented when a capacitor value of the receiving circuit has an error of 1%, and there is almost no noise.

(3) Operational amplifier: What can be seen from Fig 10 is that the synchronization can be implemented when an operational amplifier parameter of the receiving circuit has an error of 1%, and there is almost no noise.

Consequently, it is very important to select appropriate circuit components in the practical chaotic secure communication experiments. For the Lorenz system, the choice of analog multiplier is the most critical one. The sensitivity of chaotic circuit to initial value also requires that the accuracy of synchronous circuit be improved greatly. In particular, the noise may be caused due to the discretization of the parameters of the analog multipliers.

## 5. Experimental Improvement and Numerical Analysis

**Fig 11:**
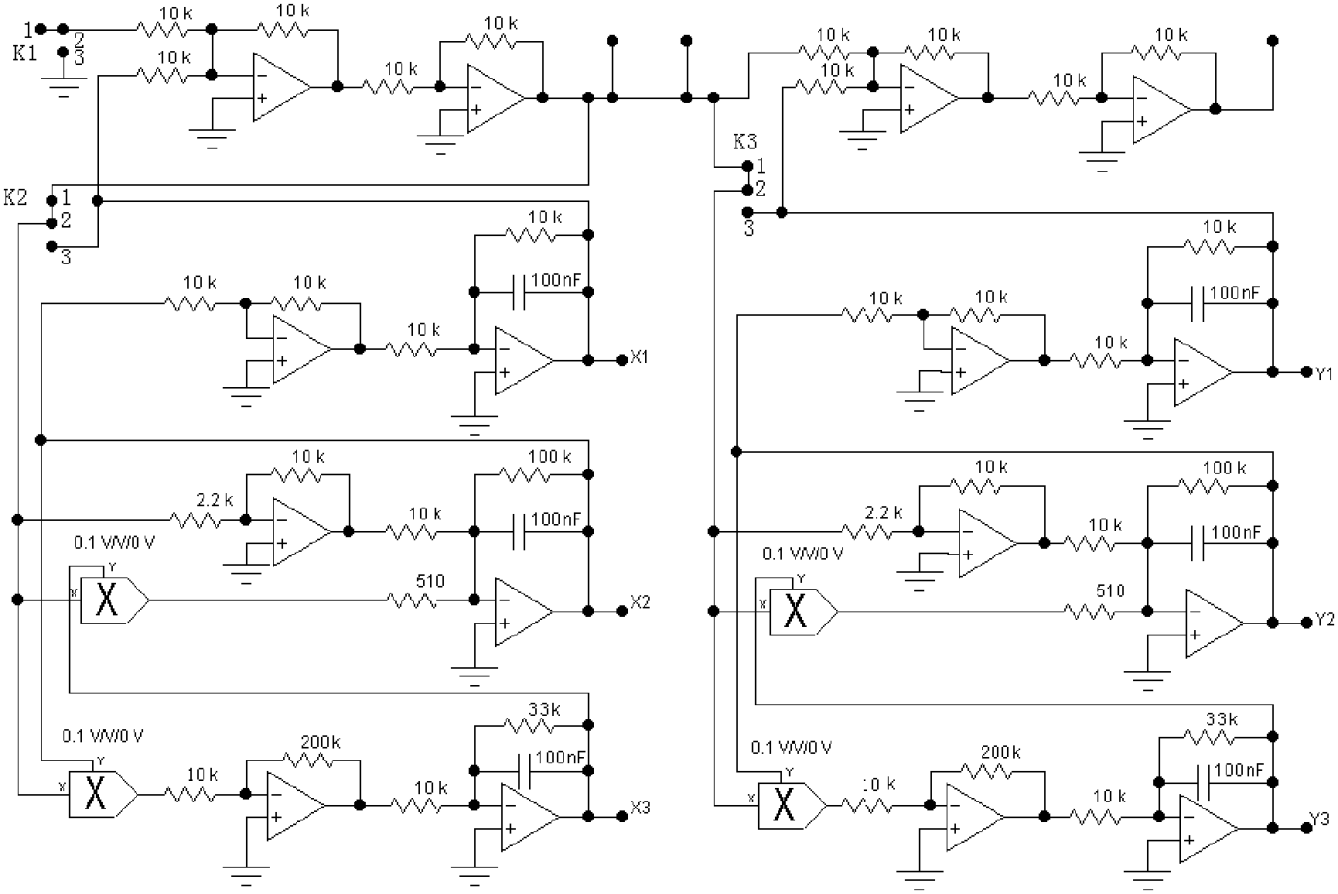
Experimental improvement circuit schematic.

In order to facilitate the experiments of chaotic synchronization, experiments of chaotic non-synchronization, experiments without signal, and experiments with signal, the following improvements are carried out to verify the comprehensive performance of the proposed scheme in the experiments. The improved chaotic secure communication circuit schematic is shown in Fig 11. The improved principles are given as follows:

1. *k*(1) is set up to control the transmitted signal *m*(*t*) with or without signal. The formula is expressed as *m*(*t*)×*k*(1). When the signal is not modulated, *k*(1) = 0. When the signal is modulated, *k*(1) = 1.
2. *k*(2) is set up to control the transmission system, which is an independent Lorenz chaotic circuit without modulation or with modulation. For Lorenz chaotic circuit without modulation, *k*(21) = 0 and *k*(23) = 1. The formula is expressed as *s*(*t*) = *x*_1_ + *m*(*t*)×*k*(1). For modulated Lorenz chaotic circuit, *k*(21) = 1 and *k*(23) = 0.
3. *k*(3) is set up to control whether the receiving system is synchronized with the transmitting system. When they are not synchronized, *k*(31) = 0 and *k*(33) = 1. At that time, the circuit is an independent Lorenz chaotic circuit, and the non-synchronization experiments can be made by two independent Lorenz circuits. When synchronized, *k*(31) = 1 and.*k*(33) = 0 The formula is expressed as *n*(*t*) = s(*t*)×*k*(31)+*x*_2_×*k*(33).

Therefore, the complete formula can be described as follows:

$$\left\{ \begin{array}{l} s(t)=x_{1}+m(t)\times k(1),\\ {\dot{x}}_{1}=-10x_{1}+10y_{\text{1}},\\ {\dot{y}}_{1}=45s(t)-y_{1}-19.6s(t)z_{1},\\ {\dot{z}}_{1}=20s(t)y_{1}-3z_{1}, \end{array}\right.$$ (17)

And

$$\left\{ \begin{array}{l} n(t)=s(t)\times k(31)+x_{2}\times k(33)\\ {\dot{x}}_{2}=-10x_{2}+10y_{2}\\ {\dot{y}}_{2}=45n(t)-y_{2}-19.6n(t)z_{2}\\ {\dot{z}}_{2}=20n(t)y_{2}-3z_{2} \end{array}\right.$$ (18)

**Fig 12:**
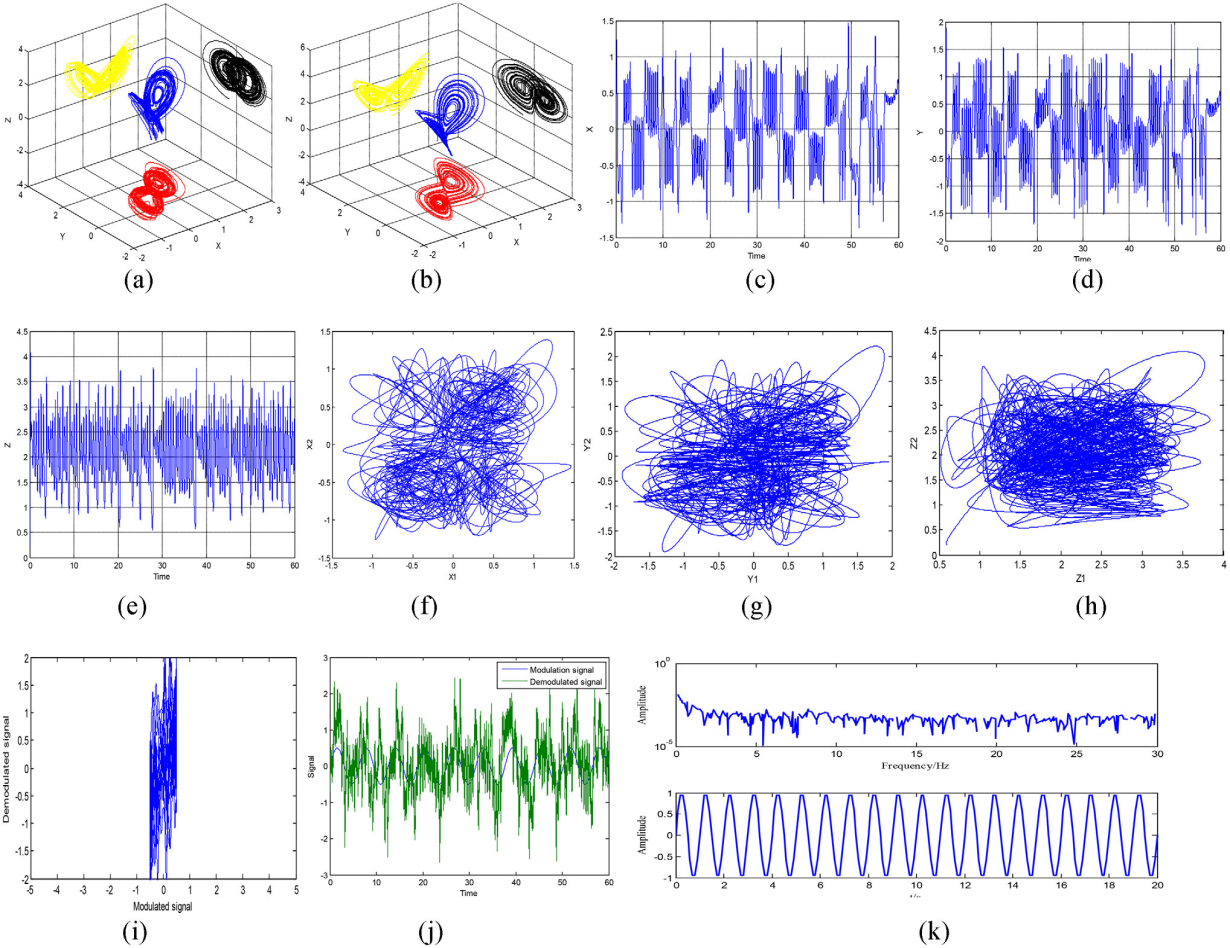
Simulation results. **(a)** attractors for transmitter, **(b)** attractors for receiver, **(c) *x*** waveform, **(d)** *y* waveform, **(e)** *z* waveform, **(f-h)** non-synchronization phase diagram, **(i)** modulation-demodulation phase diagram, **(j)** modulation signal and demodulation signal, **(k)** the power spectrum of the transmitted signal and the recovered signal.

In the following, Matlab simulation results are shown in Fig 12. Fig 12(a) and 12(b) show the chaotic attractors of the transmitter and the receiver. The waveforms for the state variables of *x*, *y*, and *z* are given in Fig 12(c), 12(d) and 12(e). Fig 12(f), 12(g) and 12(h) show the non-synchronization phase diagrams of *x*_1_*x*_2_,*y*_1_*y*_2_, and *z*_1_*z*_2_. Fig 12(i) shows the modulation and demodulation phase diagram. Fig 12(j) shows the modulation signal and the demodulation signal waveforms. The frequency spectrum of the transmitted signal and the recovered signal are given in Fig 12(k). What can be seen from Fig 12(k) is that the spectrum of the transmitted signal is fully embedded into the chaotic signal spectrum, and the transmitted signal is recovered after synchronization.

## 6. Hardware Implementation

In this paper, the proposed Lorenz chaotic secure communication circuit is characterized by its high accuracy and good robustness. In order to verify this conclusion, a new master-slave chaotic modulation communication circuit is tested. The actual circuit diagram is shown in Fig 11. The key to a successful circuit experiment and high or low output indicator is the consistency of the corresponding components of the transmitting circuit and the receiving circuit. The standard error is defined as follows:

$$R_{e}=\sqrt{\frac{\sum_{i=1}^{n}{\left(e_{si}-e_{ri}\right)}^{2}}{n}},$$ (19)

where *e* is the measurement parameter of resistor and capacitor. The subscript *s* and *r* respectively represent the transmitting end and the receiving end, and *i* is a label. Then *R*_*e*_ represents the total error of the circuit parameters. Here, the operational amplifiers are considered as ideal components.

*K*_1_: When the modulation signal is not added, the modulation signal input of the transmitting system needs to be grounded in order to eliminate interference. At this point, the 2–3 terminal of *K*_1_ is connected, and it is changed to the 1–2 terminal coupled with the signal.

*K*_2_: It is not only the circuit control of the transmitting system, but also the experimental control of synchronization and non-synchronization. When the 2–3 terminal is connected, the transmitting system constitutes an independent Lorenz unit circuit. When the 1–2 terminal is connected, the transmitting system is connected with the modulation circuit.

*K*_3_: It is not only the circuit control of the receiving system, but also the experimental control of synchronization and non-synchronization. When the 2–3 terminal is connected, the receiving system constitutes an independent Lorenz unit circuit. When the 1–2 terminal is connected, the receiving system is connected with the modulation circuit.

**Fig 13:**
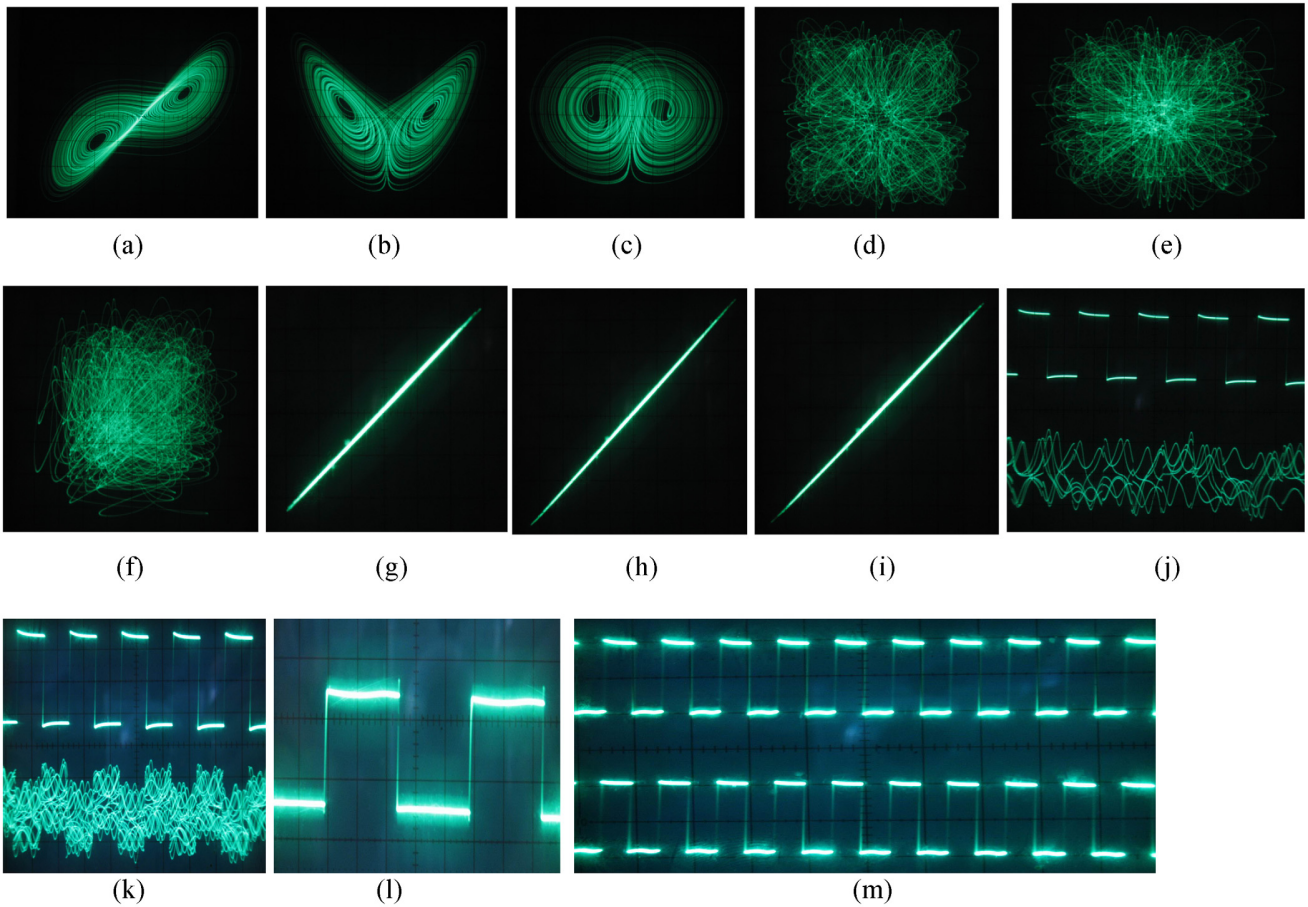
Chaotic secure communication circuit signal photos. **(a) *xy*** phase diagram, **(b)** *xz* phase diagram, **(c)** *zy* phase diagram, **(d-f)** non-synchronization phase diagrams, **(g-i)** synchronization phase diagrams, **(j)** signal and channel waveform, **(k)** signal and channel waveform, **(l)** modulation-demodulation signal amplification, **(m)** transmitting and receiving waveform.

**Fig 14:**
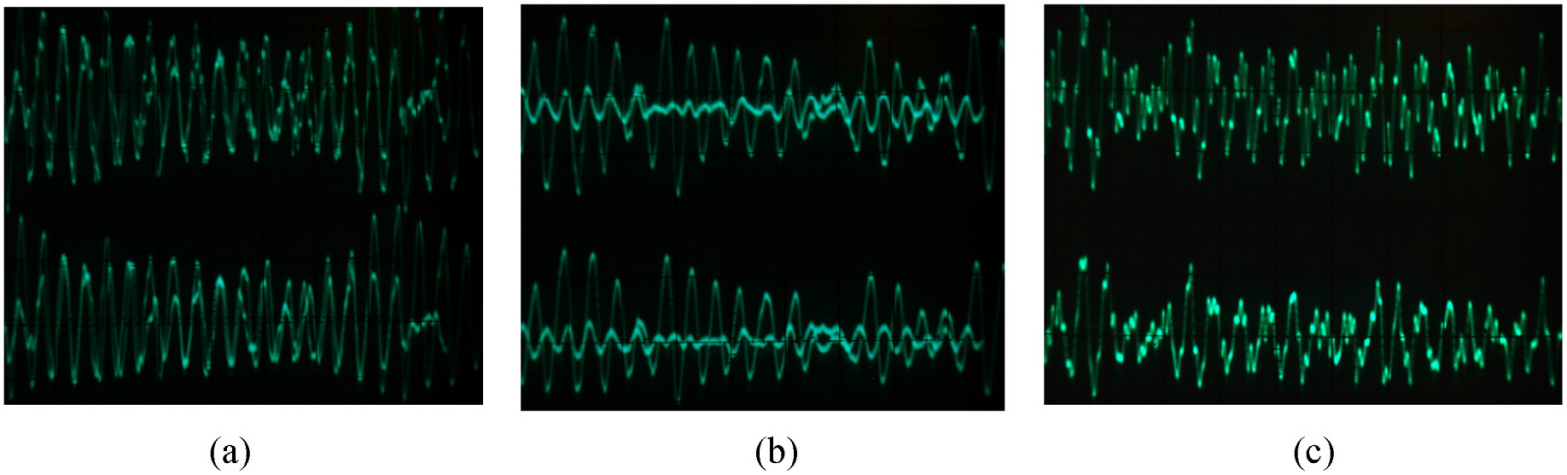
The transmitter input modulation signal and the receiver demodulation output signal.

**Fig 15:**
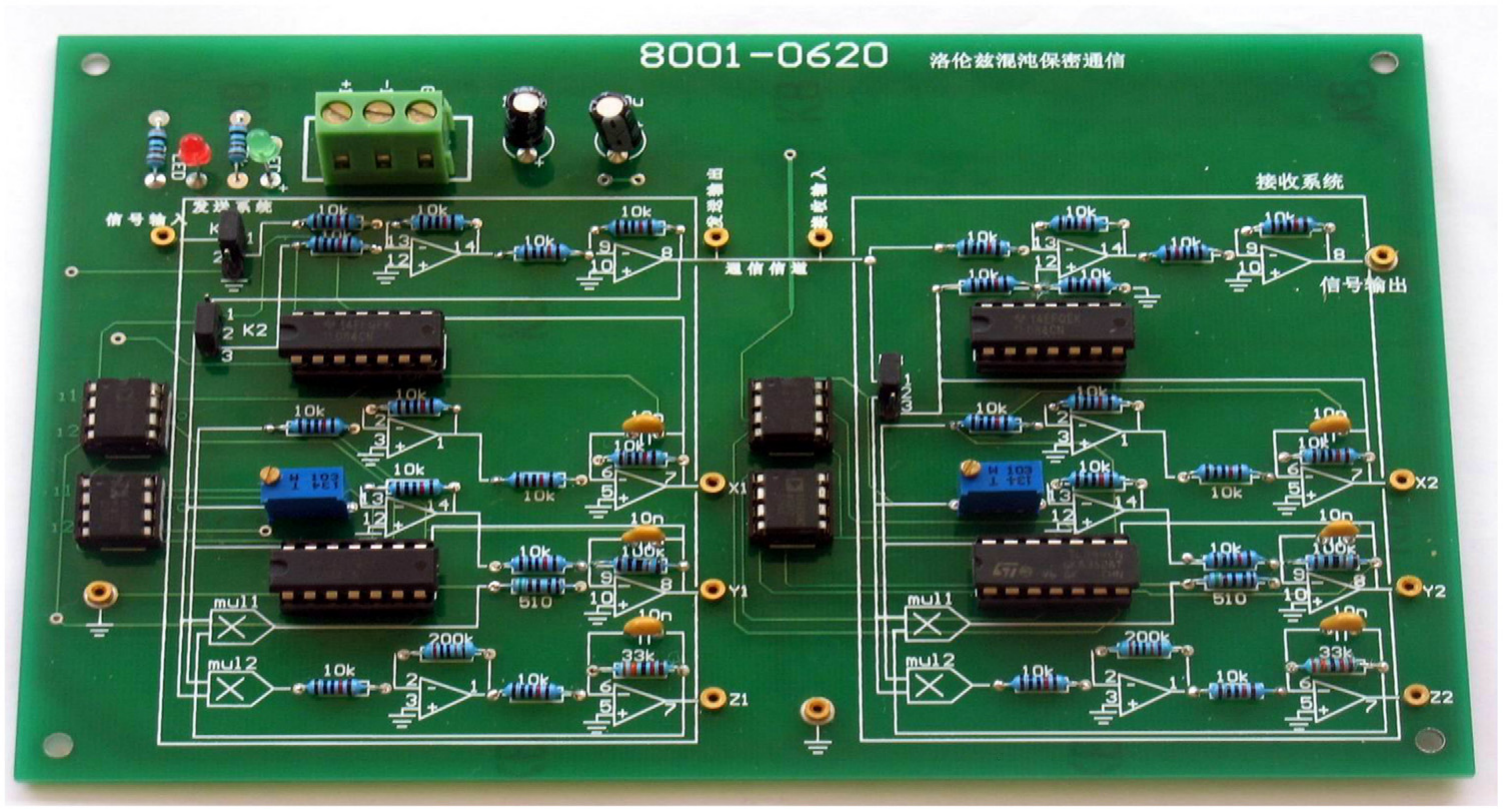
Experimental circuit board photo.

In the following, the actual hardware circuit is built to make experiment measurements according to Fig 11. As long as the circuit components are selected carefully, the circuit can be achieved as desired. An oscilloscope is used to measure the circuit. The displayed photos of the actual experiment circuit are shown in Fig 13. Fig 13(a), 13(b) and 13(c) show the phase diagrams of three output variables. The non-synchronization phase diagrams of the receiver and the transmitter are shown in Fig 13(d), 13(e) and 13(f). The synchronization phase diagrams of the receiver and the transmitter are shown in Fig 13(g), 13(h) and 13(i). Fig 14(a), 14(b) and 14(c) show the modulation signal of the transmitter and the demodulation signal of the receiver after accessing the radio. What is found is that the speech signal is basically demodulated by the receiver. That is to say, the secure reception is implemented. However, the effect of the speech communication is not perfect because the multipliers with discrete parameters produce relatively large synchronization noises. The experimental circuit board photo is shown in Fig 15.

From the experimental results, what can be concluded is that the synchronization effect of the Lorenz chaotic circuit is robust. Taking into account the magnitude of the chaotic signal is a voltage magnitude, the noise coefficient of the circuit is *δ*_*N*_ < 10^−5^. What can be seen from Fig 13(m) is that the amplitude of the modulation signal is 10 mV. The distortion degree of *δ*_*N*_ < 10^−2^ is measured by oscilloscope using the same method. Several actual measurement circuit boards were welded carefully, and all the experimental results and the index were repeated. Such measurement method and experimental results have not been reported to date; therefore, it is of great practical significance.

However, the proposed chaotic secure communication method still has certain limitation. On one hand, because traditional amplifiers and current conveyors have frequency limitations, they have limited performance in implementing nonlinear circuits. On the other hand, it is well known that most of the chaotic system can generate finite chaotic attractors. However, some evidences have confirmed that the chaotic system composed of multi-scroll attractor shows more complex dynamical behaviors. Therefore, nonlinear oscillators composed of multi-scroll chaotic attractor show more complicate and rich chaotic dynamics. And they are often used for generating complex secure keys and carrying wave for chaotic secure communication or image encryption [18, 19]. That is, it is more reliable to use chaotic system composed of multi-scroll attractors to implement chaotic secure communication or image encryption. Moreover, another issue is how to improve the unpredictability of the chaotic communication system. Those designs can be enhanced if the chaotic oscillator possesses more positive Lyapunov exponents, because it determines the unpredictability grade of the chaotic oscillator [45, 46]. Therefore, using chaotic system composed of multi-scroll attractors to achieve secure communication will be the next problem to be addressed.

## 7. Conclusion

In this paper, a novel approach is presented to enhance the security performance of transmission signal and improve the vulnerability of chaotic modulation. For the first time, the improved Lorenz chaotic system in a simple chaotic modulation method is implemented to illustrate the heightening of security in communication. In this research, the receiving system is easier to maintain good synchronization with the transmitting system by using the Lorenz chaotic optimization circuit, and the robustness of the proposed secure communication scheme was validated. Another advantage of the proposed scheme guarantees an effective inspection of the comprehensive performance of the circuit by improving the experimental circuit and measurement method. Moreover, in order to verify the strength limit of the transmitted signal, the characteristic of being broadband and the required accuracy for the electronic components, some numerical simulations are presented by Multisim. The simulation results verify that the proposed scheme can implement the effective transmission and reception of the signals. The intensity of per transmitted signal strength must be far less than that of the chaotic signal in order to achieve the effective transmission and reception of the signal. What can be seen from the simulation results and the experimental results is that the comprehensive performance of the proposed scheme seems to be satisfactory for the chaotic secure communication applications. More specifically, the debugging difficulty of Lorenz chaotic secure communication circuit is much larger than that of Chua's circuit. In future, this scheme will be verified for its’ application into other chaotic systems.
